# HHLA2 deficiency inhibits non‐small cell lung cancer progression and THP‐1 macrophage M2 polarization

**DOI:** 10.1002/cam4.4081

**Published:** 2021-06-21

**Authors:** Wenjie Sun, Shuying Li, Guiliang Tang, Shaoxing Sun, Yuan Luo, Rui Bai, Linzhi Han, Xueping Jiang, Yanping Gao, Zhengrong Huang, Junhong Zhang, Yan Gong, Conghua Xie

**Affiliations:** ^1^ Department of Radiation and Medical Oncology Zhongnan Hospital of Wuhan University Wuhan China; ^2^ Department of Biological Repositories Zhongnan Hospital of Wuhan University Wuhan China; ^3^ Tumor Precision Diagnosis and Treatment Technology and Translational Medicine Hubei Engineering Research Center Zhongnan Hospital of Wuhan University Wuhan China; ^4^ Hubei Key Laboratory of Tumor Biological Behaviors Zhongnan Hospital of Wuhan University Wuhan China; ^5^ Hubei Cancer Clinical Study Center Zhongnan Hospital of Wuhan University Wuhan China

**Keywords:** HHLA2, M2 polarization, non‐small cell lung cancer, tumor‐associated macrophages, tumorigenesis

## Abstract

**Background:**

Human endogenous retrovirus‐H long terminal repeat‐associating protein 2 (HHLA2) is a member of B7 family, which is upregulated in multiple tumors. However, its exact functions in non‐small cell lung cancer (NSCLC) have not been fully understood. This study aimed to investigate the biological roles of HHLA2 in human NSCLC and the relevant mechanisms. In addition, the effects of tumor cell‐derived HHLA2 on tumor‐associated macrophage (TAM) polarization were explored.

**Methods:**

NSCLC cell growth, migration, and invasion were assessed by colony formation and modified Boyden chamber assays. Cell cycle and the CD163+ TAMs were examined by flow cytometry. A co‐culture model of THP‐1 macrophages and NSCLC cells was conducted to investigate the impacts of tumor cell‐derived HHLA2 on THP‐1 macrophage polarization. Moreover, a xenograft nude mouse model was established to explore the effects of HHLA2 on tumorigenesis in vivo.

**Results:**

HHLA2 was upregulated in A549 and H1299 cells compared with the normal lung epithelial BEAS‐2B cells. HHLA2 deficiency inhibited NSCLC cell proliferation, migration, invasion, and induced G0/G1 phase arrest partially via inhibiting EGFR/MAPK/ERK signaling pathway. Furthermore, HHLA2 knockdown inhibited M2 polarization of TAMs via downregulating IL‐10. In addition, knockdown of HHLA2 inhibited tumor growth in vivo.

**Conclusion:**

HHLA2 downregulation inhibited NSCLC growth and TAM M2 polarization. HHLA2 may serve as a therapeutic target and promising prognostic biomarker in NSCLC.

## Graphical abstract

HHLA2 deficiency inhibited the proliferation, migration, invasion, and induced G0/G1 arrest of A549 and H1299 cells by inactivating EGFR/MAPK/ERK signaling pathway. In addition, knockdown of HHLA2 in NSCLC cells inhibited M2 polarization of THP‐1 macrophages via decreasing the secretion of IL‐10.

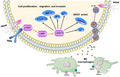

## 1 INTRODUCTION

Lung cancer is one of the most common malignant cancers worldwide and bears the highest mortality rate of cancer.^1^, ^2^ Approximately 80%–85% of lung cancer cases are non‐small cell lung cancer (NSCLC).^3^ Although significant advances have been made in diagnostic modalities, most of NSCLC patients are discovered at the advanced stages. Several therapeutic methods, including surgical intervention, radiotherapy, chemotherapy, and targeted therapy have been used to treat NSCLC in clinic. However, the prognosis is not ideal, as the overall cure and 5‐year survival rate of NSCLC remain low.^4^, ^5^ The mechanisms of NSCLC evolution and progression are complex, which involve numerous genetic and epigenetic alternations. Therefore, discovery of genetic biomarkers and molecular mechanisms of NSCLC is crucial for the development of novel diagnostic and therapeutic strategies.

Human endogenous retrovirus‐H Long repeat‐associating 2 (HHLA2, also known as B7H5/B7H7/B7y), as a new member of B7 family, was reported to be overexpressed in multiple human cancers, such as lung cancer, triple‐negative breast cancer, osteosarcoma, and renal cell cancer.^6^, ^7^, ^8^, ^9^, ^10^, ^11^ However, it shows limited expression in normal tissues. Numerous studies revealed that overexpression of HHLA2 might be involved in tumor progression.^7^, ^8^, ^9^, ^10^, ^11^, ^12^ Recent researches focused more on the immunomodulatory function of HHLA2. HHLA2 can provide a co‐stimulatory signal to induce T cell activation or a co‐inhibitory signal to inhibit T cells, depending on the receptors it binds.^13^, ^14^, ^15^ Zhu *et al* indicated that HHLA2 stimulated T cell proliferation and cytokine production via binding to TMIGD2 on antigen‐presenting cells.^14^ However, Zhao *et al* demonstrated that HHLA2 inhibited T cell proliferation and reduced cytokine production, thus to suppress T cell‐mediated antitumor responses.^13^

Tumor‐associated macrophages (TAMs), developed from monocytes, have been regarded as key mediators of tumorigenesis.^16^, ^17^, ^18^ TAMs are always divided into two types: (I) classically activated macrophages (M1), which secrete pro‐inflammatory cytokines and exhibit pro‐inflammatory functions; (II) alternatively activated macrophages (M2), which release anti‐inflammatory cytokines and exhibit anti‐inflammatory functions.^19^, ^20^ In the process of tumor development, TAMs, especially M2 TAMs, are recruited into tumors, promoting tumor growth, invasion, metastasis, and angiogenesis.^21^, ^22^, ^23^ In addition, TAMs can inhibit immunity, induce immune tolerance, and suppress responses to standard‐of‐care therapeutics.^21^, ^24^, ^25^, ^26^ Therefore, inhibition of M2 TAM infiltration or polarization has attracted increasing attention in cancer treatment. Previous studies revealed that HHLA2 was constitutively expressed in human monocytes.^27^ Immature monocytes migrated to tumor tissues and developed into TAMs. Qi *et al* found that TAMs were significantly higher in the HHLA2 low expression group in malignant glioma, indicating that HHLA2 participated in the process of monocyte development into TAMs.^28^

Many B7 family members were reported to function as oncogenes and promote epithelial‐to‐mesenchymal transition (EMT) in NSCLC.^29^, ^30^ Previous study demonstrated that HHLA2 modulated malignant behaviors in clear cell renal cell carcinoma (ccRCC).^31^ Several studies suggested that HHLA2 participated in the regulation of T cell function. However, its biological function in NSCLC and involvement in TAM polarization remains unclear.

In this study, we investigated the expression pattern and the function of HHLA2 in NSCLC and provided evidence that knockdown of HHLA2 inhibited NSCLC proliferation, migration, invasion, and M2 polarization of TAMs. In addition, the potential possible mechanism was investigated.

## 2 MATERIALS AND METHODS

### 2.1 Data download and analysis

Corresponding data about gene expression RNAseq in pan‐cancer were downloaded from the UCSC Xena database. We extracted the data of HHLA2 expression and then drew a picture to describe the expression pattern of HHLA2 in pan‐cancer using R package.

### 2.2 Cell culture

Human normal lung epithelial cell line (BEAS‐2B), five human NSCLC cell lines (A549, H1299, H1975, H460, and PC9), and human monocytic cell line (THP‐1) were purchased from the American Type Culture Collection (ATCC, Shanghai, China). All the cells were cultured in RPMI‐1640 medium (Gibco, USA) or Dulbecco's modified Eagle's medium (Gibco, USA) containing 10% fetal bovine serum (FBS, Gibco), and 1% penicillin/streptomycin (Sigma, USA) under the conditions of 37°C and 5% CO_2_.

### 2.3 Quantitative real‐time polymerase chain reaction (qRT‐PCR)

Total RNA was extracted using TRIzol (Vazyme, China) according to the manufacturer's instructions. After examining the RNA concentration and purity using a NanoDrop spectrophotometer (Thermo Scientific, USA), 1 μg of RNA was reversely transcribed to cDNA using a Reverse Transcriptase Kit (Vazyme, China). Then, qRT‐PCR was performed in a 20 μl reaction volume containing 10 μl of ChamQ^TM^ SYBR^®^ qPCR Master Mix (Vazyme, China), 1 μl of primers, 1 μl of cDNA, and 8 μl of RNase‐free water. GADPH was used as an internal reference. The relative mRNA expression was quantified using 2^−ΔΔCt^ method. The primer sequences are listed in Table 1.

**TABLE 1:** Primers used for qRT‐PCR

Gene	Forward primer (5ʹ−3ʹ)	Reverse primer (5ʹ−3ʹ)
CD80	GCAGGGAACATCACCATCCA	TCACGTGGATAACACCTGAACA
CD86	CTGCTCATCTATACACGGTTACC	GGAAACGTCGTACAGTTCTGTG
CD163	AAAAAGCCACAACAGGTCGC	CTTGAGGAAACTGCAAGCCG
CD206	TGAATTGTACTGGTCTGTCCT	CTGTGGTGCTGTGCATTTATCT
Arg−1	CTTGGCAAAAGACTTATCCTTAG	ATGACATGGACACATAGTACCTTTC
CCL18	TCTATACCTCCTGGCAGATTC	TTTCTGGACCCACTTCTTATTG
IL−10	TTAAGGGTTACCTGGGTTGC	CTGGGTCTTGGTTCTCAGCTT
HHLA2	AGTGGTGCTAAAGGTGGGAGTT	CATGTTGTTTTCAGAGATAGGTGTGT
GAPDH	TGGAAGGACTCATGACCACA	TTCAGCTCAGGGATGACCTT

### 2.4 Immunoblotting

Proteins were extracted using RIPA lysate (Beyotime) containing 1% protease inhibitors and 1% phosphatase inhibitors (Sigma). The protein concentration was quantified using the BCA kit (Beyotime). Then, equal amounts of protein samples were separated by SDS‐PAGE (Bio‐Rad) and transferred to the polyvinylidene fluoride membrane (Millipore). After blocked with 5% nonfat milk at room temperature for 2 h, the membranes were incubated with primary antibodies at 4°C overnight. The primary antibodies used were listed in Table 2. After washing with TBST (10 min ×4), the membranes were then incubated with corresponding secondary antibodies at room temperature for 1 h. After washing with TBST again (10 min ×4), protein signals were detected by an enhanced chemiluminescence (ECL) detection system (Bio‐Rad).

**TABLE 2:** Antibodies used for immunoblotting

Target	Manufacturer	Catalog number	Dilution factor
HHLA2	ABclonal	A13262	1:1000
GAPDH	Proteintech	10494–1‐AP	1:1000
P27	Proteintech	25614–1‐AP	1:1000
CDK2	Proteintech	10122–1‐AP	1:1000
CDK4	Proteintech	11026–1‐AP	1:1000
CDK6	Proteintech	14052–1‐AP	1:1000
CyclinD1	Proteintech	26939–1‐AP	1:1000
CyclinE1	Proteintech	11554–1‐AP	1:1000
E‐Cadherin	ABclonal	A3044	1:1000
N‐Cadherin	ABclonal	A19083	1:1000
Vimentin	ABclonal	A19607	1:1000
MMP2	ABclonal	A19080	1:1000
MMP9	ABclonal	A2095	1:1000
p‐EGFR	Cell Signaling Technology	2220S	1:1000
t‐EGFR	Proteintech	66455–1‐lg	1:1000
p‐MEK	Cell Signaling Technology	#9154	1:1000
t‐MEK	Proteintech	11049–1‐AP	1:1000
p‐ERK1/2	Cell Signaling Technology	#4370	1:1000
t‐ERK1/2	Proteintech	16443–1‐AP	1:1000
IL−10	Affinity	DF6894	1:1000

### 2.5 RNA interference

To silence the expression of HHLA2 in A549 and H1299 cells, three specific small interfering RNAs targeting human HHLA2 (siHHLA2) and negative control (NC) were designed and synthesized by GenePharma Technologies (Shanghai). A total of 3 × 10^5^ cells were seeded into each well of 6‐well plates. After culturing for 24 h, siHHLA2 or NC was transfected into the cells with jetPRIME^®^ transfection reagent (Polyplus‐transfection^®^ SA, France) according to the manufacturer's protocol. The sequences of the designed oligonucleotides against HHLA2 and NC were listed in Table 3.

**TABLE 3:** Oligonucleotides sequences of NC and siHHLA2

	Sense(5'−3’)	Antisence(5'−3’)
NC	UUCUCCGAACGUGUCACGUTT	ACGUGACACGUUCGGAGAATT
siHHLA2#1	GCAUAUUCCCUUUGGCUUUTT	AAAGCCAAAGGGAAUAUGCTT
siHHLA2#2	GCUAUAAGGUUCACAGUUATT	UAACUGUGAACCUUAUAGCTT
siHHLA2#3	CCUUCUGGACGAAGGAAUUTT	AAUUCCUUCGUCCAGAAGGTT

### 2.6 Cell counting kit‐8 (CCK8) assay

Transfected cells were adjusted to 3 × 10^4^/ml and plated into 96‐well plates (3000 cells/well in 100 µL medium). After culturing for 24, 48, 72, and 96 h, the supernatant was replaced with 100 µL of serum‐free medium added with 10 µL of CCK8 (Dojindo, Japan) and incubated at 37°C in the dark for 1 h. Subsequently, the absorbance value of each well at 450 nm was measured by a microplate reader (Rayto). Each group had five wells and the experiment was repeated three times.

### 2.7 Colony formation assay

After transfection for 48 h, transfected cells were harvested, and seeded into 6‐well plates at a density of 800 cells/well in 2 mL of culture medium. Then, the cells were cultured at 37°C in 5% CO_2_ for 2 weeks. Afterward, the colonies were fixed with 4% paraformaldehyde (PFA) for 30 min and stained with 0.1% crystal violet for 30 min. The number of colonies was then counted manually under a microscope (Nikon Microphot‐FX).

### 2.8 Cell cycle analysis

After washing twice with cold phosphate‐buffered saline (PBS), the cells were performed with a cell cycle staining kit (MultiSciences Biotech) according to the manufacturer's instructions. The cell cycle profiles were assayed on a FACScan flow cytometer (Beckman).

### 2.9 Wound healing assay

Transfected cells were seeded into 6‐well plates. When reached 100% confluency, the cells were scratched straight with a 10 μl pipette tip and washed with PBS gently to remove cell debris. After that, 2 mL of serum‐free medium was added into each well and the pictures were taken by phase‐contrast microscope (Nikon Microphot‐FX) at 0, 24, and 48 h. Wound healing rate (%) = (Distance of initial scratch − Distance of final imaged without cells) / Distance of initial scratch.

### 2.10 Modified Boyden chamber migration and invasion assays

Cell migration and invasion assay were performed using the modified Boyden chamber with a pore size of 8 μm (Corning) in 24‐well plates. For the cell migration assay, approximately 4 × 10^4^ cells suspended in 200 μl of serum‐free medium were seeded in the upper compartment, 600 μl of medium containing 10% FBS was added in the lower compartment. After 24 h, the migrated cells were fixed with 4% PFA for 30 min and stained with 0.5% crystal violet for 1h, and non‐migrating cells on the upper compartment were carefully wiped off by a cotton swab. The cells were pictured by a microscope (Nikon Microphot‐FX) and counted in five random fields using ImageJ.

For cell invasion assays, 100 μl of Matrigel (Becton‐Dickinson) was added into the upper compartment and incubated for 4 h at 37°C. A total of 6 × 10^4^ cells were seeded in the upper compartment in a similar protocol.

### 2.11 Immunofluorescence

Cells transfected with siHHLA2 or NC were cultured on 24 × 24 mm glass slides. After 24 h, cells were washed three times with PBS and then fixed with 4% PFA at room temperature for 30 min. For intracellular staining of Ki‐67, the cells were penetrated with 0.5% Triton X−100 (Beyotime) for 15 min. Next, the cells were blocked in 5% bovine serum albumin at room temperature for 1 h. After incubation with the primary antibodies against Ki67 (Proteintech, 27309–1‐AP, 1:100) and ZO‐1 (Proteintech, 21773–1‐AP, 1:100) at 4°C overnight, the slips were washed with PBST and incubated with FITC‐labeled secondary antibodies at room temperature for 1 h. The nuclei were labeled with 4,6‐diamidino‐2‐phenylindole (Beyotime). Finally, images were taken by a fluorescence microscope (Olympus).

### 2.12 THP‐1 differentiation and co‐culture system

To be differentiated into adherent macrophages, THP‐1 cells (5 × 10^5^/well) were seeded into 6‐well plates in the presence of 100 ng/ml of phorbol 12‐myristate 13‐acetate (PMA, Sigma) for 24 h. The differentiated macrophages were named as M0 macrophages. To establish a co‐culture system, we transfected A549/H1299 cells with siHHLA2 or NC. At 48 h post‐transfection, we used the culture supernatant to incubate the M0 macrophages. After 48 h of co‐culture, the macrophages were harvested for analysis.

### 2.13 Flow cytometry

Single‐cell suspensions were collected and stained with FITC‐conjugated CD11b and PE‐conjugated CD163 antibodies (Biolegend) at 4°C for 40 min. Then, the cells were washed in PBS, and stained with PC5.5‐conjugated 7AAD (Biolegend) at 4°C for 5 min to eliminate dead cells. Finally, the proportion of CD163+ TAMs was examined and analyzed on a FACScan flow cytometer (Beckman).

### 2.14 Enzyme‐linked immunosorbent assay (ELISA)

The supernatants of different groups of A549 and H1299 cells were collected. The levels of IL‐10 were determined by Human ELISA Kit (Bio‐Swamp) according to the manufacturer's instructions. Each group had three wells and the experiment was repeated three times.

### 2.15 Xenograft nude mice model

BALB/c nude mice at 5 weeks were purchased from Vital River Laboratory Animal Technology Co. Ltd, and housed under specific‐pathogen‐free conditions. Approximately 1 × 10^7^ normal or HHLA2‐deficient NSCLC cells in 100 μl of PBS were implanted subcutaneously into the right armpits of nude mice. The tumor volume (0.5 × length × width^2^) was measured every other day. The mice were anesthetized with pentobarbital and imaged using IVIS Lumina XRMS Series III (PerkinElmer) at week 5. After that, mice were sacrificed and tumors were fixed for next analysis.

### 2.16 Immunohistochemistry

Harvested tumor tissues were fixed in 4% PFA, embedded in paraffin, and sectioned into 4‐μm slides. Then the slides were deparaffinized with xylene, rehydrated with graded ethanol, blocked with 3% H_2_O_2_, and followed by heat‐induced antigen retrieval. After that, the slides were incubated with primary antibodies against HHLA2 (ABclonal, A13262, 1:100), Ki67 (Proteintech, 27309–1‐AP, 1:100), N‐Cadherin (ABclonal, A19083, 1:100), and E‐Cadherin (ABclonal, A3044, 1:100) at 4˚C overnight. The next day, the slides were incubated with corresponding secondary antibodies at 37°C for 60 min. Finally, the sections were counterstained with hematoxylin, dehydrated, covered, and visualized. The slides were observed using light microscopy (Olympus, Japan) and all images were acquired at 400× magnification.

### 2.17 Statistical analysis

GraphPad Prism 7 was employed for statistical analysis. All data were presented as mean ± standard deviations (SD). The differences between two or more groups were determined by Student's *t*‐test or one‐way analysis of variance. *p* values < 0.05 were considered as statistical significance.

## 3 RESULTS

### 3.1 HHLA2 was upregulated in NSCLC cells

**FIGURE 1:**
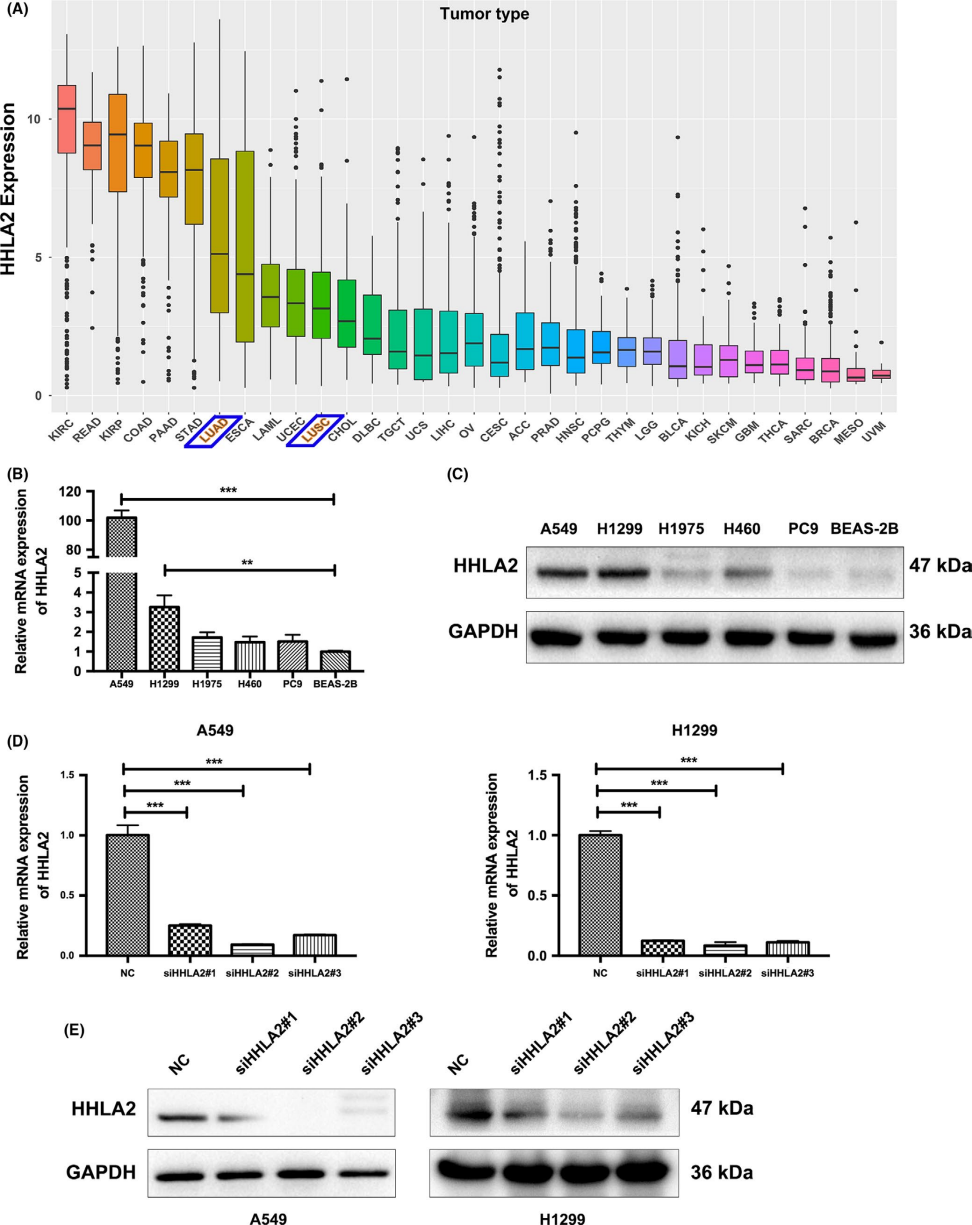
Relative expression and knockdown efficiency of HHLA2 in NSCLC cells. (A) Data from the UCSC Xena database indicated that HHLA2 were highly expressed in NSCLC. (B, C) The mRNA and protein levels of HHLA2 in NSCLC and normal lung epithelial cells. (D) qRT‐PCR analysis indicated that HHLA2 expression was significantly decreased in A549 and H1299 cells transfected with siHHLA2 compared with NC. (E) Immunoblotting analysis confirmed the knockdown efficiency. **p < 0.01, ***p < 0.001

In order to reveal the expression pattern of HHLA2 in various cancers, we downloaded RNAseq data of pan‐cancer from the UCSC Xena database. HHLA2 was highly expressed in lung adenocarcinoma and squamous cell carcinoma (Figure 1A). The mRNA and protein levels of HHLA2 in normal BEAS‐2B and five NSCLC cell lines were examined. A549 and H1299 had significantly increased HHLA2 expression compared with BEAS‐2B (Figure 1B, C). To further study the roles of HHLA2 in NSCLC tumorigenesis and progression, A549 and H1299 cells were transfected with siHHLA2 or NC. The results of qRT‐PCR and immunoblotting confirmed that HHLA2 mRNA and protein levels were decreased with siHHLA2 (Figure 1D, E). Since siHHLA2#2 and siHHLA2#3 were more effective in both A549 and H1299 cell lines, they were used for the following experiments.

### 3.2 Knockdown of HHLA2 inhibits NSCLC cell proliferation

**FIGURE 2:**
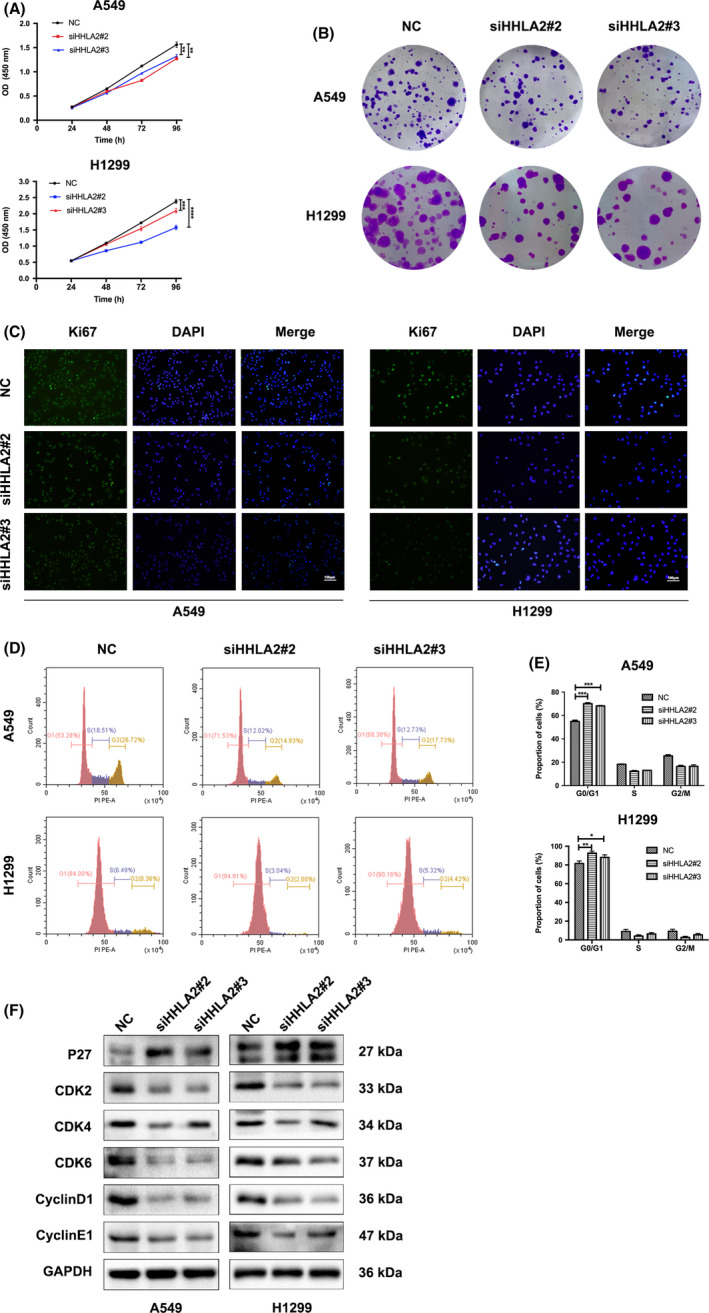
Knockdown of HHLA2 inhibited NSCLC cell proliferation and cell cycle progression. (A) CCK8 assay revealed that HHLA2 silencing inhibited cell proliferation in A549 and H1299 cells. (B) Colony formation assay indicated that knockdown of HHLA2 reduced the numbers of colonies 14 days after siHHLA2 transfection. (C) Immunofluorescent staining indicated that Ki67 was downregulated with HHLA2 knockdown. Scale bar: 100 μm. (D, E) HHLA2 silencing induced G0/G1 phase arrest in A549 and H1299 cells. (F) The cell cycle‐related proteins were measured with immunoblotting. *p < 0.05, **p < 0.01, ***p < 0.001

CCK8 and colony formation assays were performed to investigate the effects of HHLA2 on cell proliferation. CCK8 assay showed that A549 and H1299 cell viability was decreased with HHLA2 silencing (Figure 2A). Consistent with the CCK8 results, cell colonies in the siHHLA2 group were significantly less than those in the NC group (Figure 2B). In addition, immunofluorescence indicated that knockdown of HHLA2 downregulated Ki67 (Figure 2C).

We further explored the effects of HHLA2 on the cell cycle. Flow cytometry results indicated that knockdown of HHLA2 blocked the cell cycle at the G0/G1 phase (Figure 2D, E). The protein levels of CyclinD1/E1 and CDK2/4/6 were decreased, while the protein levels of P27 were increased with HHLA2 knockdown (Figure 2F).

### 3.3 Knockdown of HHLA2 inhibited NSCLC cell migration and invasion

**FIGURE 3:**
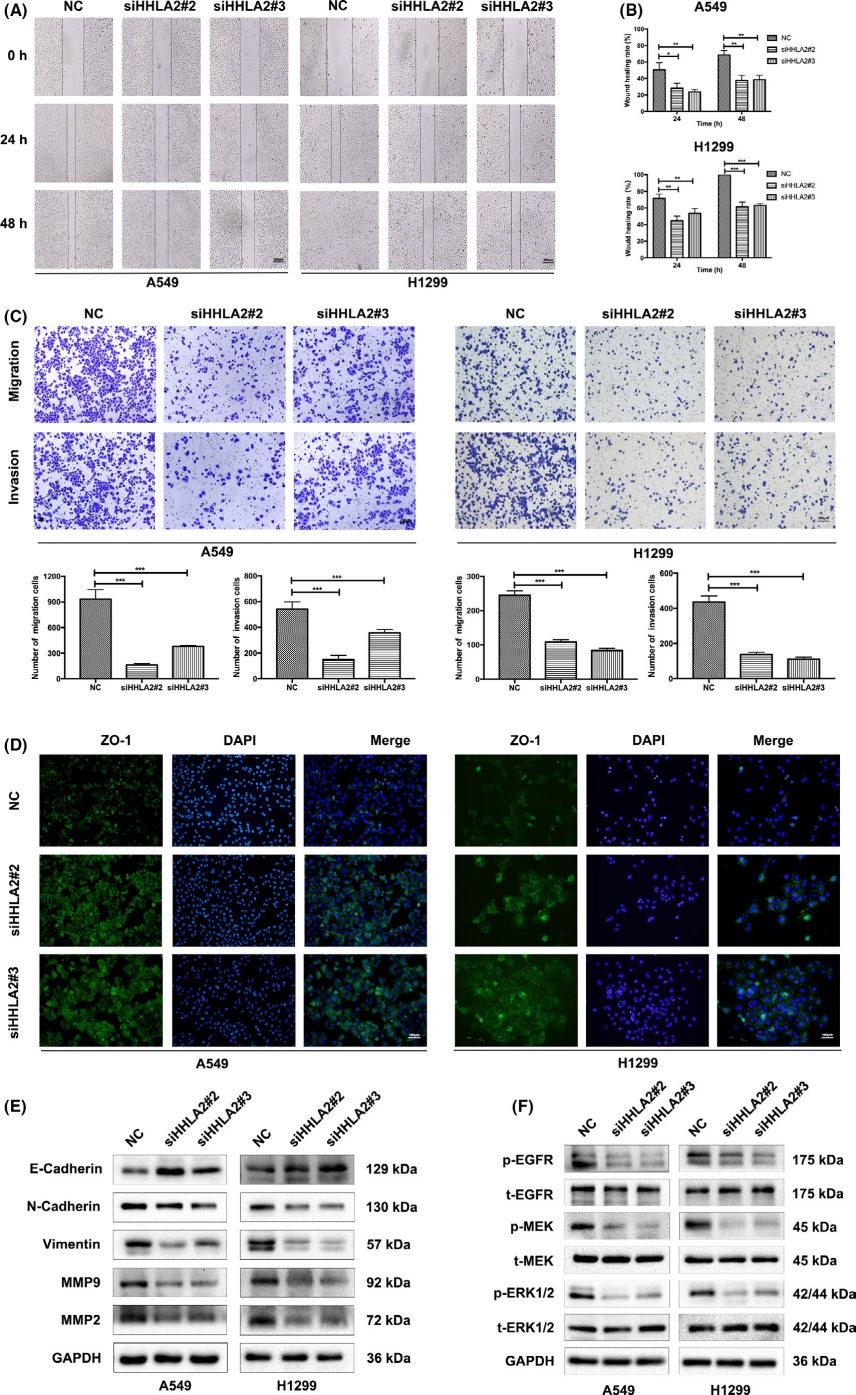
Knockdown of HHLA2 inhibited NSCLC cell migration, invasion via modulating the EMT‐related proteins. (A, B) HHLA2 knockdown decreased wound healing rates of A549 and H1299 cells. Scale bar: 200 μm. (C) Modified Boyden chamber assay revealed that HHLA2 deficiency inhibited the migration and invasion of A549 and H1299 cells. Scale bar: 200 μm. (D) Immunofluorescent analysis indicated that the expression of ZO‐1 was upregulated in HHLA2‐deficient A549 and H1299 cells. Scale bar: 100 μm. (E) Immunoblotting was performed to evaluate the protein levels of E‐Cadherin, N‐Cadherin, Vimentin, MMP2, and MMP9 expression after HHLA2 knockdown. (F) Knockdown of HHLA2 inhibited the activity of EGFR/MAPK/ERK signaling pathway in A549 and H1299 cells. *p < 0.05, **p < 0.01, ***p < 0.001

Cell migration and invasion were measured by the wound healing and modified Boyden chamber assays. The wound healing rates of A549/H1299 cells were significantly decreased with siHHLA2 (Figure 3A, B). In addition, modified Boyden chamber assay showed that the number of migrated and invaded cells was obviously decreased with HHLA2 knockdown (Figure 3C). Immunofluorescence of ZO‐1 indicated that knockdown of HHLA2 enhanced the cell–cell attachment (Figure 3D). HHLA2 deficiency also upregulated E‐Cadherin, but downregulated N‐Cadherin, Vimentin, MMP2, and MMP9 in A549 and H1299 cells (Figure 3E). Taken together, the results indicated that HHLA2 silencing inhibited the migration and invasion of NSCLC cells via modulating the EMT‐related proteins.

Since EGFR/MAPK/ERK signaling pathway participates in the evolution and progression of various tumors, we examined the phosphorylation levels of ERK1/2, MEK, and EGFR. The protein levels of p‐ERK1/2, p‐MEK, and p‐EGFR were significantly decreased in the A549/H1299 cells transfected with siHHLA2, while there was no difference in the expression of t‐ERK1/2, t‐MEK, and t‐EGFR between siHHLA2 group and NC group (Figure 3F). These results suggested that knockdown of HHLA2 inactivated EGFR/MAPK/ERK pathway.

### 3.4 Knockdown of HHLA2 in NSCLC cells inhibited M2 polarization of THP‐1 macrophages

**FIGURE 4:**
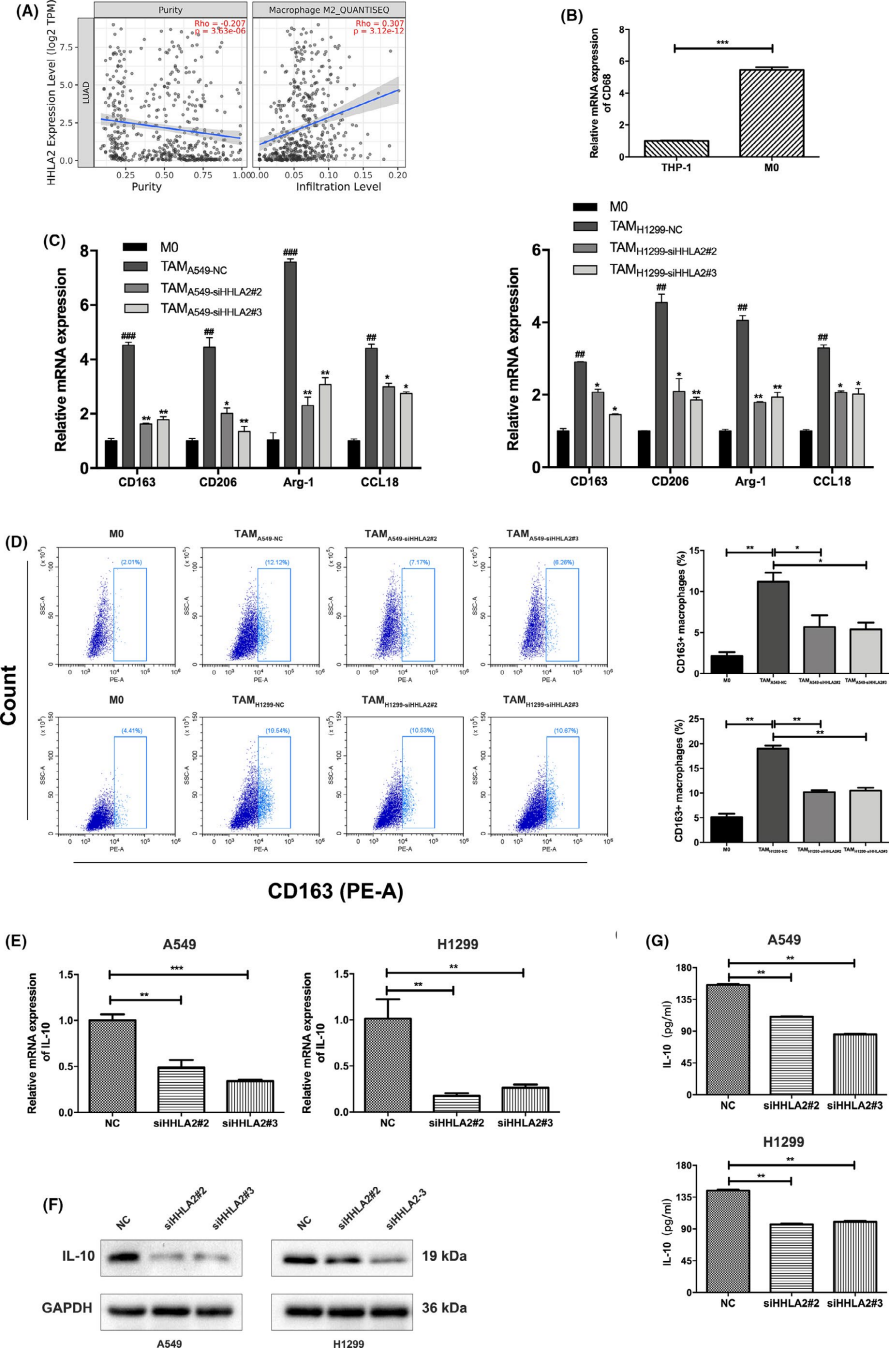
Knockdown of HHLA2 in A549 and H1299 cells inhibited M2 polarization of THP‐1 macrophages. (A) TIMER database indicated a positive correlation between M2 macrophage infiltration and HHLA2 expression in lung adenocarcinoma. (B) CD68 was upregulated in M0 macrophages compared with THP‐1 cells. (C) M2 macrophage marker expression in M0 macrophages and co‐cultured TAMs. ^#^ means the significant difference between TAM_A549‐NC_/ TAM_H1299‐NC_ and M0 macrophages. * means the significant difference between TAM_A549‐siHHLA2_/TAM_H1299‐siHHLA2_ and TAM_A549‐NC_/TAM_H1299‐NC_. (D) Flow cytometry revealed that knockdown of HHLA2 in A549 and H1299 cells decreased CD163+ macrophages. (E) IL‐10 mRNA levels in A549 and H1299 cells were transfected with NC or siHHLA2. (F) Protein levels of IL‐10 in A549 and H1299 cells were transfected with NC or siHHLA2. (G) The secretion of IL‐10 in the supernatant in A549 and H1299 cells was transfected with NC or siHHLA2. *p < 0.05, **p < 0.01, ***p < 0.001; ^##^ *p* < 0.01, ^###^ *p* < 0.001

A positive correlation between M2 macrophage infiltration and HHLA2 expression in lung adenocarcinoma was identified in TIMER database (Figure 4A). THP‐1 cells were induced from suspension to adherent growth after incubation with PMA. The mRNA levels of macrophage marker CD68 were upregulated (Figure 4B). To explore whether knockdown of HHLA2 in NSCLC cells was involved in the process of THP‐1 macrophage polarization, we transfected A549/H1299 cells with siHHLA2 or NC. The M0 macrophages were then incubated with conditional medium from A549/H299‐NC and A549/H299‐siHHLA2 cells for 48 h. Untreated M0 macrophages were used as the control group. M2 macrophage markers CD163, CD206, Arg‐1, and CCL18 were upregulated in the TAM_A549‐NC_ and TAM_H1299‐NC_ groups compared with the M0 group. After treatment with A549‐siHHLA2 or H1299‐siHHLA2 supernatant, the mRNA levels of CD163, CD206, Arg‐1, and CCL18 of TAMs were markedly downregulated (Figure 4C). Moreover, we measured the proportion of CD163+ macrophages by flow cytometry. The results indicated that knockdown of HHLA2 in A549 and H1299 cells decreased CD163+ macrophages (Figure 4D). These results suggested the inhibition of M2 polarization of TAMs by HHLA2 deficiency.

To explore the underlying mechanisms, we analyzed the related cytokines that influenced TAM polarization. The results of qRT‐PCR, immunoblotting, and ELISA revealed a marked decrease of IL‐10 in siHHLA2‐transfected A549 and H1299 cells (Figure 4E–G). These results suggested that knockdown of HHLA2 in NSCLC inhibited TAM M2 polarization via downregulating IL‐10.

In order to examine the interaction between ERGR/MAPK/ERK activation and IL‐10/M2 polarization of THP‐1 macrophages, we blocked ERK pathway using U0126 in A549 and H1299 cells, and found that IL‐10 secretion and M2 polarization of THP‐1 macrophages were unaffected. In addition, IL‐10 blockade had no role in the phosphorylation of ERK1/2. These results suggested that ERGR/MAPK/ERK activation and IL‐10/M2 polarization upon HHLA2 silencing were parallel (Supplemental Figure).

### 3.5 Knockdown of HHLA2 suppressed tumorigenesis in vivo

**FIGURE 5:**
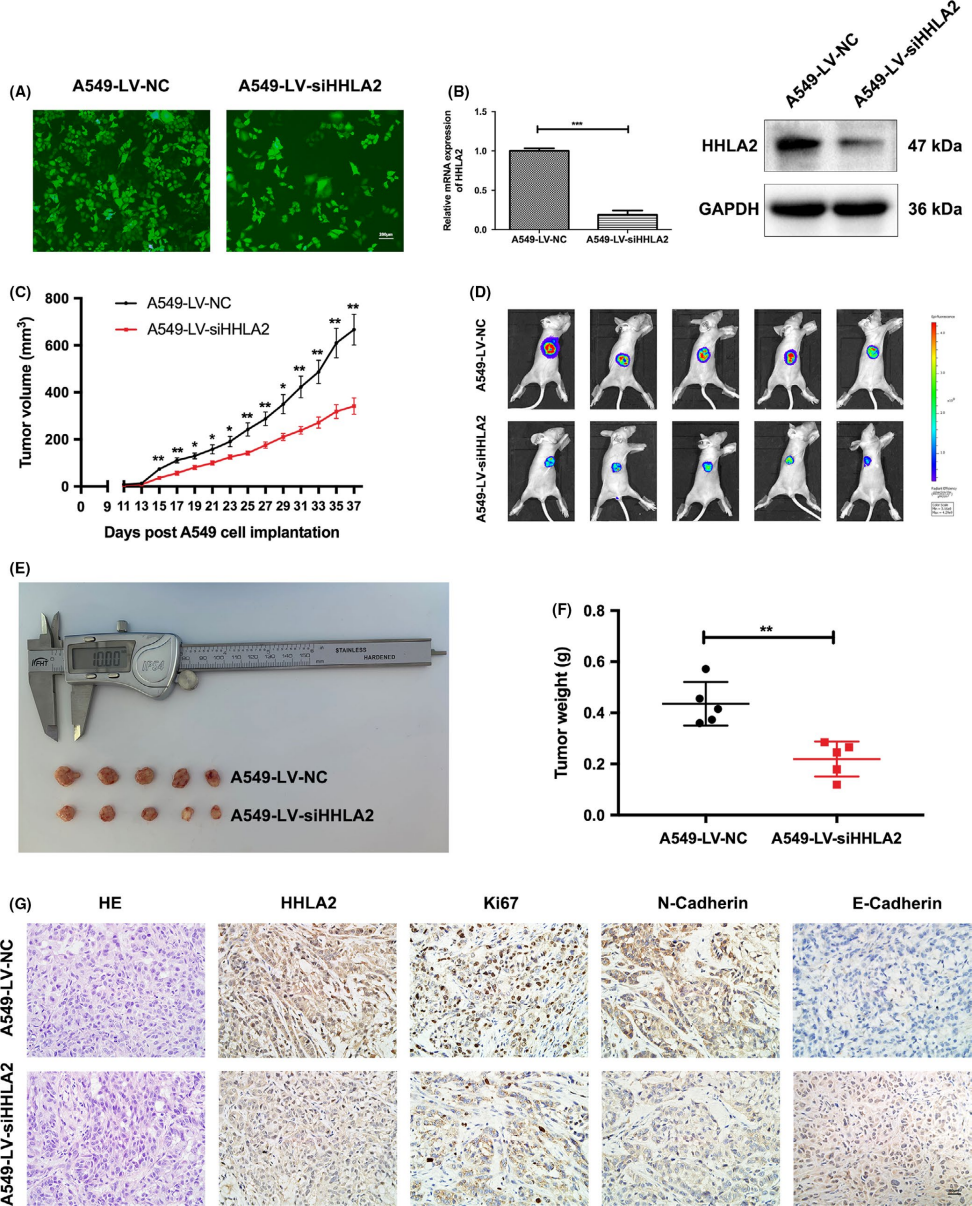
Knockdown of HHLA2 inhibited tumorigenesis in vivo. (A) Representative fluorescence micrographs of A549 cells after transfected with LV‐NC and LV‐siHHLA2 labeled with GFP. Scale bar: 200 μm. (B) mRNA and protein levels of HHLA2 in the A549‐LV‐siHHLA2 group were significantly lower than those in the A549‐LV‐NC group. (C) Xenotransplantation tumor growth curve. (D‐F) Sizes and weight of tumor mass. (G) Representative immunohistochemistry staining micrographs of HHLA2, Ki67, N‐Cadherin, and E‐Cadherin in the xenograft tumors. Scale bar: 50 μm. *p < 0.05, **p < 0.01, ***p < 0.001

A tumor xenograft model was established by implanting HHLA2‐knockdown A549 cells into BALB/c nude mice. Fluorescent microscopy indicated that lentiviruses were successfully transfected into A549 cells (Figure 5A). qRT‐PCR and immunoblotting indicated that LV‐siHHLA2 strongly inhibited the expression of HHLA2 (Figure 5B). The in vivo experiments revealed that mice injected with LV‐siHHLA2 cells yielded a smaller tumor size (Figure 5C–E). The tumor weight was lower in the A549‐LV‐siHHLA2 group (Figure 5F). Immunohistochemistry results showed that HHLA2, Ki67, and N‐Cadherin were significantly reduced in the A549‐LV‐siHHLA2 group, while the expression of E‐Cadherin was increased in the A549‐LV‐siHHLA2 group (Figure 5G).

## 4 DISCUSSION

HHLA2 was identified in 1999 as a molecule containing immunoglobulin‐like domains.^32^ Recently, it was revealed that HHLA2 shares 23%–33% similarity in amino acid sequence with other B7 family members, and it was identified as a new member of B7 family.^13^ HHLA2 is a multifunctional protein that is abnormally expressed in various cancers. It was reported that HHLA2 promoted the progression of several human tumors, such as lung cancer, breast cancer, and oral cancer.^8^, ^9^, ^33^ Previous studies focused more on its regulation on T cells. The relationship between HHLA2 expression and TAM infiltration is still debatable. In this study, we focused more on the biological roles of HHLA2 in NSCLC cells and the immunomodulatory effects of HHLA2 on macrophage polarization.

HHLA2 has been shown to be upregulated in ccRCC and knockdown of HHLA2 inhibited the malignant behaviors of ccRCC cells.^31^ In this work, the biological roles of HHLA2 in NSCLC were investigated. The expression levels of HHLA2 were increased in NSCLC cells compared with normal lung epithelial cells. It was demonstrated for the first time that knockdown of HHLA2 inhibited NSCLC cells proliferation, migration, and invasion via modulating EGFR/MAPK/ERK signaling pathway. During EMT, epithelial cells lose cell–cell adhesion, acquiring a mesenchymal phenotype to be migratory and invasive.^34^, ^35^ In the current study, our results indicated that NSCLC cell migration and invasion were suppressed by HHLA2 knockdown via regulating EMT‐related proteins.

TAMs are important drivers of tumor development and progression.^36^, ^37^ Recently, investigations on macrophage polarization have attracted great attention. HHLA2 protein is constitutively expressed on the surface of human monocytes or macrophages.^13^, ^14^ The tumor‐infiltrating immune cell model showed that HHLA2 was negatively associated with TAMs in malignant glioma, indicating that the importance of association between HHLA2 and TAMs.^28^ Through establishing a co‐culture system of THP‐1 macrophages and NSCLC cells, we observed a significant decrease in the expression of M2 macrophage markers; and an increase of M1 macrophage markers of TAMs after co‐cultured with supernatant of HHLA2‐deficient NSCLC cells. These results suggested that the knockdown of HHLA2 in NSCLC cells inhibited M2 polarization of TAMs.

Previous studies reported that HHLA2 was widely expressed in patients with PD‐L1‐negative NSCLC, intrahepatic cholangiocarcinoma, and osteosarcoma, suggesting that HHLA2 might be a promising immunotherapy target.^10^, ^38^, ^39^ In addition, HHLA2 was more prevalent than PD‐L1 in intrahepatic cholangiocarcinoma and osteosarcoma.^10^, ^39^ In this study, we found that in addition to be an inhibitory checkpoint in NSCLC, HHLA2 also contributed to tumor progression via directly inhibiting malignant behaviors and regulating M2 polarization of TAMs. Collectively, these findings suggested that HHLA2 played multiple‐level antitumor roles as a potential therapeutic target for NSCLC.

Our study has several limitations. Previous studied revealed that HHLA2 was highly expressed in lung cancer,^8^, ^38^ more clinical samples of NSCLC patients should be used to validate our results. HHLA2 is expressed in primates but absent in rodents,^9^, ^13^, ^40^ it is necessary to find a suitable animal model to perform further researches. In addition, gain‐of‐function assays with HHLA2 overexpression should be used to confirm the regulatory effects of HHLA2 on NSCLC malignant behaviors and TAM polarization.

## 5 CONCLUSION

**FIGURE 6:**
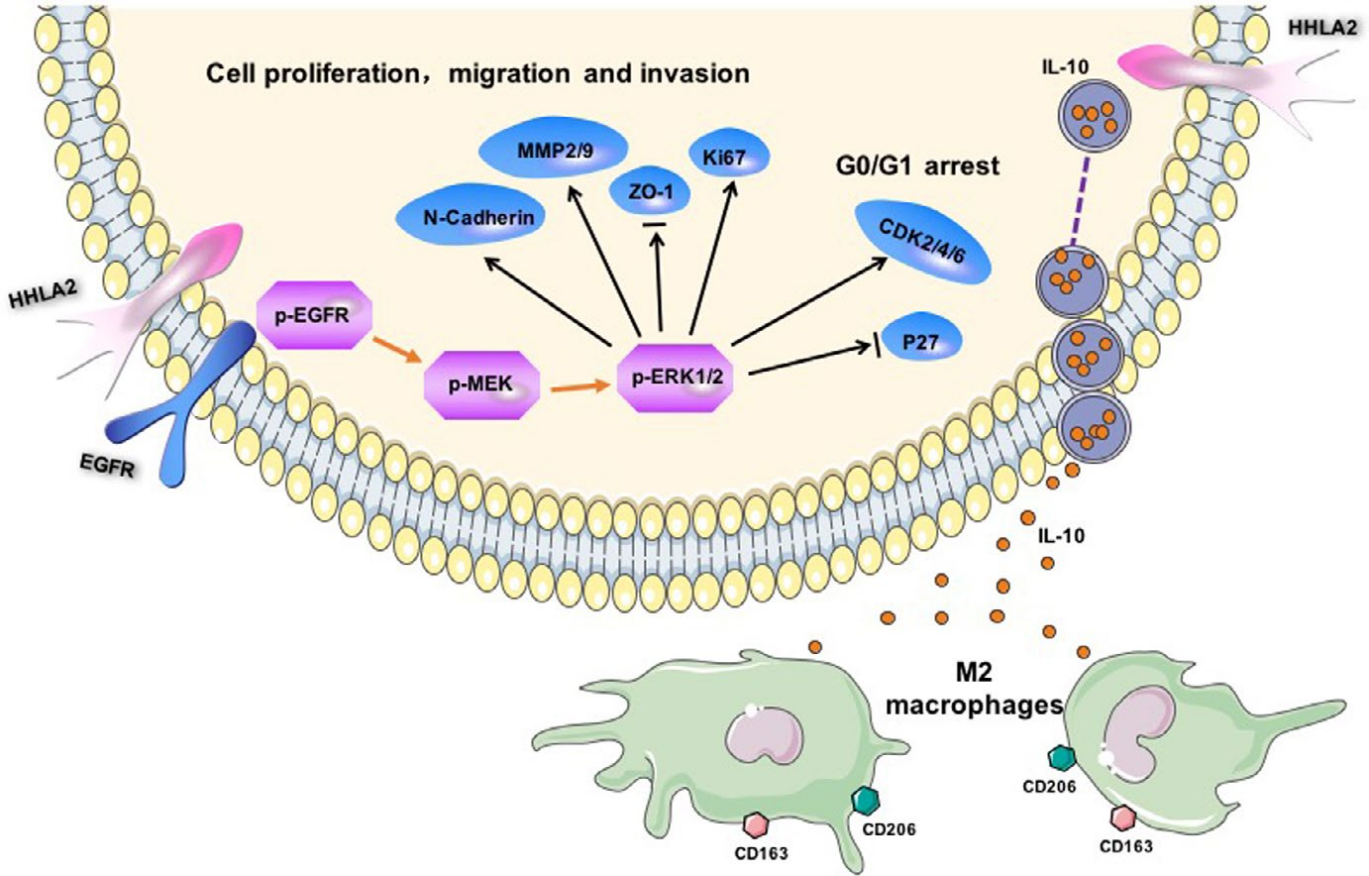
Mechanism diagram. HHLA2 deficiency inhibited the proliferation, migration, invasion, and induced G0/G1 arrest of A549 and H1299 cells by inactivating EGFR/MAPK/ERK signaling pathway. In addition, knockdown of HHLA2 in A549 and H1299 cells inhibited M2 polarization of THP‐1 macrophages via decreasing the secretion of IL‐10

As shown in Figure 6, HHLA2 deficiency inhibited the proliferation, migration, invasion, and induced G0/G1 arrest of A549 and H1299 cells by inactivating EGFR/MAPK/ERK signaling pathway. In addition, knockdown of HHLA2 in A549 and H1299 cells inhibited M2 polarization of THP‐1 macrophages via decreasing the secretion of IL‐10.

## 6 ETHICS APPROVAL AND CONSENT TO PARTICIPATE

All animal experiments were performed according to the National Institutes of Health guidelines and approved by the Institutional Animal Care and Use Committee (IACUC) of Wuhan University (WP2020‐08021).

## CONFLICT OF INTEREST

All authors declare no conflict of interest.

## AUTHORS’ CONTRIBUTIONS

Wenjie Sun, Shuying Li, Yan Gong, and Conghua Xie designed the experiments; Wenjie Sun, Shuying Li, Guiliang Tang, Shaoxing Sun, and Yuan Luo performed the experiments; Rui Bai, Linzhi Han, and Xueping Jiang prepared figures; Yanping Gao, Zhengrong Huang, and Junhong Zhang was responsible for statistical analysis and provided helpful suggestions; Wenjie Sun, Shuying Li, and Yan Gong wrote the manuscript. All authors read and approved the final version of the article.

## Supporting information

Supplementary Material

## Data Availability

All data in this study are available upon request from the corresponding author.
