# Correction to “HHLA2 deficiency inhibits non‐small cell lung cancer progression and THP‐1 macrophage M2 polarization”

**DOI:** 10.1002/cam4.7205

**Published:** 2024-05-08

Sun W, Li S, Tang G, et al. HHLA2 deficiency inhibits non‐small cell lung cancer progression and THP‐1 macrophage M2 polarization. Cancer Med. 2021;10:5256–5269. https://doi.org/10.1002/cam4.4081

A revised Figure 3 is shown below and has been added to the online version of the article.

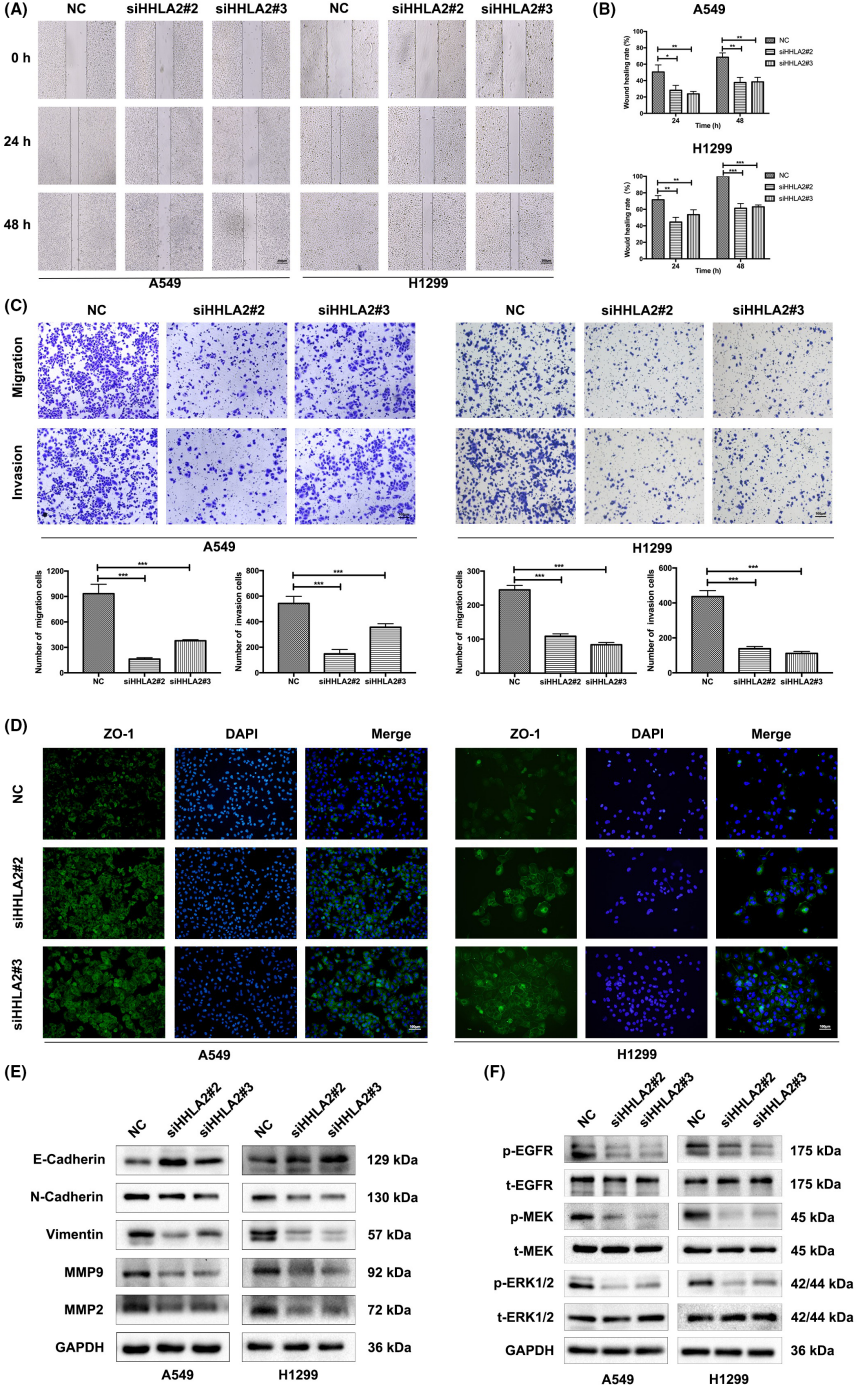

We apologize for this error.

