# Supplementary material for: HHLA2 deficiency inhibits non‐small cell lung cancer progression and THP‐1 macrophage M2 polarization
Source: Cancer Med. 2021 Jun 21;10(15):5256–69. doi: 10.1002/cam4.4081 (PMC8335813; doi:10.1002/cam4.4081)
Supplement: Supplementary file 1 — Supplementary Material [file CAM4-10-5256-s001.pdf]

Supplementary figure and legends

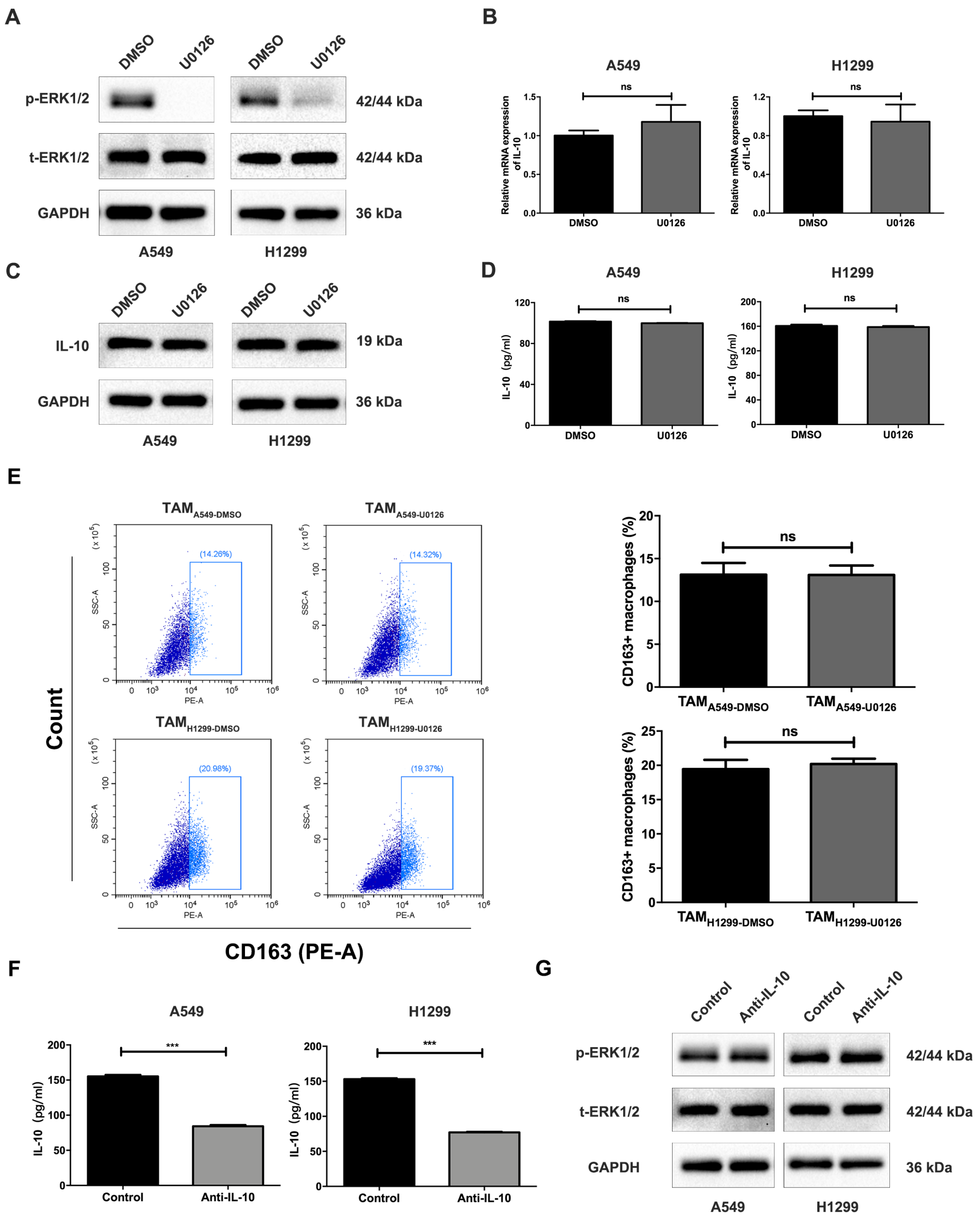

Supplementary Figure 1 ERGR/MAPK/ERK activation and IL-10/M2 polarization is parallel. (**A**) The phosphorylation of ERK1/2 were markedly inhibited after 6h of U0126 treatment. (**B, C**) The mRNA and protein levels of IL-10 in A549 and H1299 cells were unaffected after 48h of U0126 treatment. (**D**) ELISA showed that IL-10 in the supernatant of A549 and H1299 cells was unaffected after 48h of U0126 treatment. (**E**) The proportion of CD163+

macrophages incubated with the conditional medium of A549/H1299-DMSO cells and A549/H1299-U0126 cells. (**F**) ELISA showed that anti-IL-10 antibody can neutralize IL-10 in the supernatant of A549 and H1299 cells effectively. (**G**) IL-10 blockade had no role in the phosphorylation of ERK1/2. ns, not significant, ***$P < 0.001$.
